# Antithrombotic Effect of Oil from the Pulp of Bocaiúva—*Acrocomia aculeata* (Jacq.) Lodd. ex Mart. (Arecaceae)

**DOI:** 10.3390/nu16132024

**Published:** 2024-06-26

**Authors:** Isabelly Teixeira Espinoça, Denise Caroline Luiz Soares Basilio, Anna Júlia Papa de Araujo, Rafael Seiji Nakano Ota, Kamylla Fernanda Souza de Souza, Nadla Soares Cassemiro, Davi Campos Lagatta, Edgar Julian Paredes-Gamero, Maria Lígia Rodrigues Macedo, Denise Brentan Silva, Janaina de Cássia Orlandi Sardi, Danilo Wilhelm-Filho, Ana Cristina Jacobowski, Eduardo Benedetti Parisotto

**Affiliations:** 1 Faculty of Pharmaceutical Sciences, Food and Nutrition (FACFAN), Federal University of Mato Grosso do Sul (UFMS), Campo Grande 79070-900, MS, Brazil; isabellyteixeira237@gmail.com (I.T.E.); denise.carolineluiz@hotmail.com (D.C.L.S.B.); anna.julia.papa@ufms.br (A.J.P.d.A.); rafa.seiji21@gmail.com (R.S.N.O.); nadla.cassemiro@ufms.br (N.S.C.); davi_campos@ufms.br (D.C.L.); edgar.gamero@ufms.br (E.J.P.-G.); ligia.macedo@ufms.br (M.L.R.M.); denise.brentan@ufms.br (D.B.S.); anacristinaj@gmail.com (A.C.J.); 2 Department of Biochemistry, Federal University of São Paulo, São Paulo 4044-020, SP, Brazil; kamylla.bio@outlook.com; 3 Laboratory of Natural Products and Mass Spectrometry (LAPNEM), Faculty of Pharmaceutical Sciences, Food and Nutrition (FACFAN), Federal University of Mato Grosso do Sul (UFMS), Campo Grande 79080-190, MS, Brazil; 4 Dental Research Division, Guarulhos University, Guarulhos 07023-070, SP, Brazil; janasardi@gmail.com; 5 Department of Ecology and Zoology, Center for Biological Sciences (CCB), Federal University of Santa Catarina, Florianópolis 88040-900, SC, Brazil; danilowilhelmfilho@gmail.com

**Keywords:** *Acrocomia aculeata*, toxicity, cardiovascular disease, reactive oxygen species, antiaggregant, antioxidant

## Abstract

The study aimed to evaluate the antithrombotic action of *Acrocomia aculeata* pulp oil (AAPO) in natura, in an in vitro experimental model. AAPO was obtained by solvent extraction, and its chemical characterization was performed by gas chromatography coupled to a mass spectrometer (GC-MS). In vitro toxicity was evaluated with the Trypan Blue exclusion test and in vivo by the *Galleria mellonella* model. ADP/epinephrine-induced platelet aggregation after treatment with AAPO (50, 100, 200, 400, and 800 μg/mL) was evaluated by turbidimetry, and coagulation was determined by prothrombin activity time (PT) and activated partial thromboplastin time (aPTT). Platelet activation was measured by expression of P-selectin on the platelet surface by flow cytometry and intraplatelet content of reactive oxygen species (ROS) by fluorimetry. The results showed that AAPO has as major components such as oleic acid, palmitic acid, lauric acid, caprylic acid, and squalene. AAPO showed no toxicity in vitro or in vivo. Platelet aggregation decreased against agonists using treatment with different concentrations of AAPO. Oil did not interfere in PT and aPTT. Moreover, it expressively decreased ROS-induced platelet activation and P-selectin expression. Therefore, AAPO showed antiplatelet action since it decreased platelet activation verified by the decrease in P-selectin expression as well as in ROS production.

## 1. Introduction

Cardiovascular diseases are one of the main causes of death in the world, and platelets play an important role in thrombosis and atherosclerosis [1]. Platelets are small cellular fragments derived from medullary polyploid megakaryocytes responsible for controlling processes related to health and disease [2], and reactive oxygen species (ROS) play an important role in regulating their function [3]. An imbalanced ROS production and deficits of antioxidants lead to hemostatic instabilities, which are responsible for increasing the risk of developing thrombotic and cardiovascular diseases [3,4].

Platelet activity differs among various human populations, which could explain the variability in cardiovascular diseases [5]. Moreover, antiplatelet therapy presents numerous difficulties and limitations, such as drug and nutritional interactions and a narrow therapeutic window, resulting in the need to investigate antiplatelet agents with greater efficacy and safety [6,7].

Brazil has one of the greatest biodiversities in the world and may be a source of natural bioactive compounds [8]. Among the promising plants for the development of natural products is *Acrocomia aculeata* (Jacq.) Lodd. ex Mart., popularly known as bocaiúva or macaúba [9]. It is a palm tree belonging to the Arecaceae family, native to tropical regions, and prevalently occurs in South America, mainly in the Brazilian Cerrado and Pantanal [9,10].

The bocaiúva pulp has great economic, industrial, and nutritional importance [11]. Traditionally, bocaiúva pulp is consumed in natura or in the form of sweets, ice cream, and flour [11]. In addition, it is widely recognized in folk medicine for its analgesic, healing, and laxative effects [12]. The pulp oil of *A. aculeata* (AAPO) is rich in compounds with antioxidant action [8,11], such as β-carotene, tocopherols, and fatty acids, such as oleic and palmitic acid [8,12].

Several studies have already demonstrated the beneficial effects of bioactive compounds derived from *A. aculeata*, such as hypoglycemic [13], neuroprotective [14], and antioxidant effects [15,16]. However, the antiaggregant and anticoagulant action of AAPO lacks additional studies. Thus, due to its antioxidant activity, we hypothesize that AAPO may have effects on human hemostasis, and these properties were investigated by using an in vitro experimental model.

## 2. Materials and Methods

### 2.1. Obtaining the Oil from the Pulp of A. aculeata (AAPO)

Ripe fruits of *A. aculeata*, intact and without signs of contamination and/or physical damage, were collected in January 2019, the period of greatest fruiting, in Campo Grande, Mato Grosso do Sul (20°27′54°39″ S54°38′43.732″ O), under authorization from the National System for the Management of Genetic Heritage and Associated Traditional Knowledge (SISGEN; nº A0F9FD6). 

The fruit pulp was dehydrated (50 °C) in a tray dryer at an airflow of 0.5 m/s for 18 h, and AAPO was obtained by solvent extraction using the exhaust method for 48 h with petroleum ether and hexane (2:1). The extracted oil was evaporated in a rotary evaporator, placed under a nitrogen flow (30 min), kept in a desiccator for 2 h, and stored in an amber bottle, protected from light in a cool place (10 °C) until analysis [14,17]. For the tests, dimethyl sulfoxide (DMSO 0.5%) was used as a vehicle. 

### 2.2. Gas Chromatography–Mass Spectrometry (GC-MS) Analysis

AAPO was analyzed by gas chromatography (Shimadzu QP2010, Shimadzu^®^, Tokyo, Japan) coupled to a mass spectrometer (GC-MS) equipped with a COA-20i autoinjector (Shimadzu, Kyoto, Japan). The carrier gas was helium and the pressure was 79.7 kPa. The injection temperature was 250 °C, and the temperature program was the following: 60–240 °C increasing 3 °C.min^−1^, 240–310 °C increasing 15 °C.min^−1^, and 310 °C for 10 min (isothermic). An RTx-5MS capillary column (30 mm × 0.25 mm × 0.25 μm) was used, and the mass spectra were obtained by electron ionization (EI), applying the energy 70 eV. The retention indices (RIs) were calculated using C8–C40 alkane standards (Sigma-Aldrich^®^, Cotia, São Paulo, Brazil). Identification of constituents was performed by comparing the mass spectra registered with NIST, WILEY, and FFNSC libraries, and retention indices described according to the literature [18].

### 2.3. Blood Collection and Obtaining Plasma

Human blood samples (5 mL) were collected in the same proportion by venipuncture from 20 healthy men and women, aged between 18 and 40 years, with no history of bleeding or thrombosis, after prior consent. Blood collection was performed using trisodium citrate (nine parts of blood and one part of 3.8% trisodium citrate) as an anticoagulant. PRP (platelet-rich plasma) was obtained after centrifugation for 10 min at 123× *g* at room temperature, and a PPP (platelet-poor plasma) containing approximately 10,000 platelets was obtained by diluting PRP in saline. The study protocol was approved by the Ethics Committee of the Federal University of Mato Grosso do Sul (CAAE protocol No. 57842022.2.0000.0021, approval opinion No. 5.445.802), in accordance with national and international standards for research involving human subjects.

### 2.4. Toxicity Assays

#### 2.4.1. Evaluation of In Vitro Toxicity by the Trypan Blue Exclusion Test

For the experiment, 400 μL of PRP pool was incubated (37 °C for 5 min) with 5 μL of different AAPO concentrations (50, 100, 200, 400, and 800 μg/mL) and controls; positive control: Triton X100 (1%, *v*/*v*) and negative control: DMSO (0.6%). Then, 50 μL of Trypan blue (0.4%) was added to an equal volume of PRP incubated with compounds and controls. Subsequently, they were transferred to a Neubauer chamber, and the viable and non-viable platelets were quantified. The results were expressed as the mean percentage of platelet viability. All experiments were performed in triplicate, not exceeding 3 h after collection [19].

#### 2.4.2. Evaluation of In Vivo Systemic Toxicity in Galleria Mellonella Model

For the experiment, 10 μL of AAPO of the two smallest and the two largest concentrations, yielding a final amount of 0.5, 1.0, 4.0, and 8.0 µg per larvae, or controls was injected into the hemocoel of each larva through the last left proleg using a Hamilton^®^ syringe (Hamilton Inc.^®^, Reno, NV, USA). Ten larvae weighing between 0.2 and 0.3 g without signs of melanization were used per group. Saline and DMSO (100%) were used as negative and positive controls, respectively. The larvae were incubated in the dark at 37 °C, and their survival was recorded at selected intervals for 72 h, where larvae that showed no movement to touch and high levels of melanization were counted as dead [20].

### 2.5. Determination of Platelet Aggregation

Platelet aggregation was assessed by turbidimetry using a semi-automatic aggregometer (EasyAgreg 4.0, Qualiterm^®^, São Paulo, Brazil) [21]. Platelets were counted in automatic counting equipment (Sysmex XP-300, Sysmex, Kobe, Japan) and adjusted with saline solution, obtaining a value between 200,000 and 250,000 platelets/mm^3^. Aliquots of 400 and 600 μL of the PRP pool were used for aggregation with adenosine diphosphate (ADP, 30 μM; Calbiochem^®^, Burlington, ON, Canada) or epinephrine (5 μg/mL; Hipolabor^®^, Belo Horizonte, Minas Gerais, Brazil), respectively. ADP is diluted in purified distilled water. The PRP pool was pre-incubated with 5 μL of AAPO (50, 100, 200, 400, and 800 μg/mL) at 37 °C for 5 min. Aggregation was measured in percentage (%) and recorded continuously for 5 or 10 min after the addition of agonists. DMSO (0.6%) was used as a negative control, and its average aggregation percentage was assumed to be 100%. PPP was used to adjust the baseline turbidity of the sample and Ticlopidine (10 μM) as a positive control. All tests were performed in triplicate on three independent days, not exceeding 3 h after collection.

### 2.6. Evaluation of Blood Coagulation

For coagulation evaluation, prothrombin time (PT) and activated partial thromboplastin time (aPTT) were measured following the manufacturer’s guidelines (Wiener Lab^®^, Rosario, Argentina) in triplicate and using a semi-automated coagulation system (CLOTimer^®^, Quick Timer, São Paulo, Brazil). Briefly, a PPP pool was obtained after centrifugation for 15 min at 1107× *g* and 8 °C of human blood samples collected with sodium citrate (3.8%) (9:1; *m*/*v*). About 100 μL of PPP was pre-incubated with 1.3 μL of AAPO at different concentrations (50–1000 μg/mL) or controls for 5 min at 37 °C. For normal (standard) and positive controls, plasma without added vehicle and/or treatment and heparin (17 IU/mL blood) were used, respectively. DMSO (0.6%) was used as a negative control [21].

### 2.7. Platelet Activation

#### 2.7.1. Expression of Platelet Surface P-Selectin

Platelet surface P-selectin expression was determined after incubation of PRP (400 μL) pool with 5 μL of AAPO (50, 100, 200, 400, and 800 μg/mL) or DMSO (0.6%) for 5 min at room temperature. Subsequently, the samples were stimulated with ADP (30 μM) and incubated for 5 min. Activated platelets were then labeled with fluorescein isothiocyanate (FITC) mouse anti-human CD42b (5 μL) and P-selectin with phycoerythrin (PE) mouse anti-human CD62P (5 μL) and remained in the shelter of light for 15 min. Unlabeled controls, samples labeled only with CD42b-FITC, and only CD62P-PE were used for data calibration. The assay was performed on a CytoFLEX flow cytometer (CytoFLEX, Beckman Coulter^®^, Brea, CA, USA), and 10,000 events were collected; the data were analyzed in FlowJo^®^ v10.8 software (BD Life Sciences, Franklin Lakes, NJ, USA) [22].

#### 2.7.2. Assessment of Platelet Activation by Intraplatelet Content of ROS

To evaluate platelet activation by intraplatelet content of ROS, concentrations of 50, 100, 200, 400, and 800 μg/mL of AAPO were used. DMSO (vehicle, 0.6%) was used as a negative control, and hydrogen peroxide (H_2_O_2_) as a positive control. A pool of PRP (200 μL) was incubated with 2.5 μL of AAPO concentrations or controls for 5 min. ROS content was determined by fluorescence intensity at 485 nm (excitation) and 520 nm (emission) using a multimode microplate reader (Synergy™ H1, BioTek Instruments^®^, Winooski, VT, USA) after 30 min of incubation with 10 μL 2′,7′dichlorodihydrofluorescein-diacetate (DCFH-DA, 10 µM) [23].

### 2.8. Statistical Analysis

Statistical analysis was performed using multiple comparisons of analysis of variance (ANOVA), complemented by the Tukey–Kramer test, when necessary, assuming a minimum significance level of *p* < 0.05 between different concentrations of AAPO and controls. For the *G. mellonella* model, differences in survival were compared using the log-rank test. The software used was GraphPad^®^ Prism version 8.0.2.

## 3. Results and Discussion

### 3.1. Chemical Analyses of AAPO

Oilseed plants can be affected by different environmental, climatic, cultivation, harvesting, processing, and storage factors, consequently changing the chemical composition of the extracted oils [24,25]. Despite this, the main compounds observed in AAPO by GC-MS were similar to those found in the literature [26,27] and are summarized in Table 1. Oleic acid (51.25%) was predominant, followed by palmitic acid (21.51%), and other fatty acids were also observed to a minor extent, such as ethyl oleate (8.45%), lauric acid (3.58%), caprylic acid (3.09%), and squalene (2.37%).

Recently, SANT’ANA and collaborators (2023) [15] observed an oleic acid content of approximately 49.32% in AAPO, which reflected a greater total antioxidant capacity in C57Bl/6 mice [15]. In addition, the study by PERDOMO and collaborators (2015) [28] suggested that oleic acid reduces PAI-1 levels (plasminogen activator-1 inhibitor) induced by TNF-α in vascular smooth muscle cells, thereby protecting the endothelium and modulating inflammation.

In addition, pre-treatment with physiological concentrations of oleic and palmitic acid maintains glutathione (GSH) levels and protects human endothelial cells from oxidative stress [29]. Palmitic acid is a fatty acid responsible for the palmitoylation of proteins (a reversible process involving the addition of palmitic acid to specific cysteines through a thioester bond, which provides dynamic regulation of protein functions, including processes such as phosphorylation and ubiquitination) and the biosynthesis of palmitoylethanolamine, which has neuroprotective and anti-inflammatory capacity [30]. Our findings corroborate those found by COSTA and collaborators (2020), where a concentration of approximately 15.80% palmitic acid was observed in AAPO [12].

Although less prevalent, medium-chain fatty acids lauric and caprylic, as well as squalene, a triterpene present in several vegetable oils, such as soybean oil, have already been reported to have the ability to attenuate oxidative stress [31,32,33]. Furthermore, a previous study demonstrated a concentration of approximately 31.2 mg/100 g of ascorbic acid, 46.9 mg/100 g of β-carotene, and 12.6 mg/100 g of α-tocopherol in the pulp oil of *A. aculeata* [14].

### 3.2. In Vitro and In Vivo Toxicity

The panoply of bioactive compounds present in different plant products may impact positively or negatively human and animal health [34]. The hematopoietic system is one of the most sensitive targets for toxic substances [34]; for this reason, the in vitro and in vivo toxicological assays have been performed to establish the safety criteria, quality, and efficacy, as well as to select the suitable concentrations for their therapeutic use [35].

**Figure 1:**
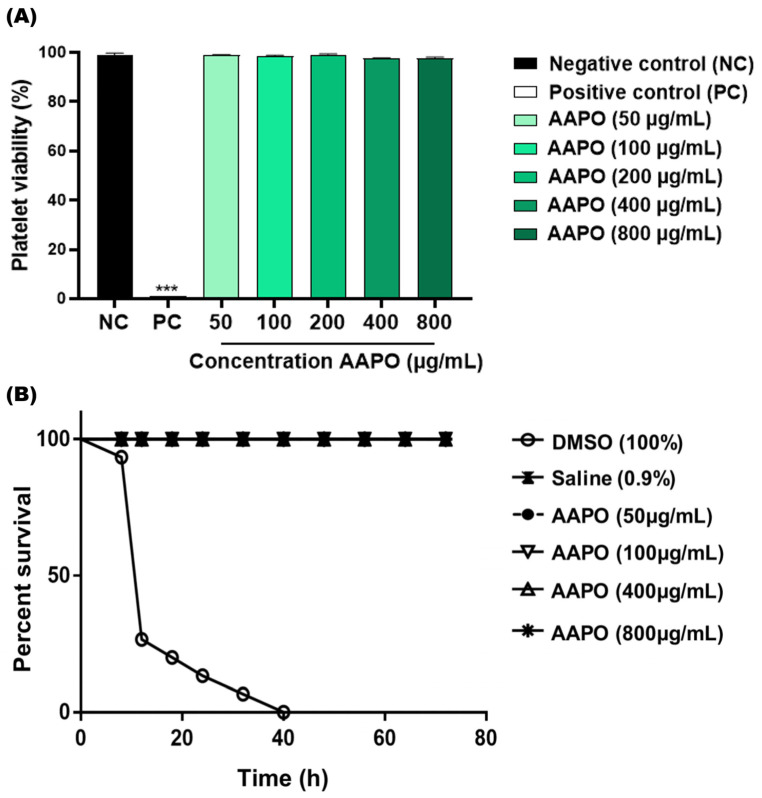
Toxicity in human platelets and systemic in *G. mellonella*. (**A**) Percentage (%) of human platelet viability obtained in PRP treated with *Acrocomia aculeata* (AAPO) pulp oil at different concentrations (50, 100, 200, 400, and 800 μg/mL); negative control (NC): vehicle (DMSO, 0.6%) and positive control (PC): Triton X100 (1%). (***) indicates statistical difference with *p* < 0.001 compared to NC. (**B**) In vivo systemic toxicity in *G. mellonella* model treated with different concentrations (50, 100, 400, and 800 μg/mL) of AAPO. The percentage of survival was evaluated for 72 h; negative control (NC): saline and positive control: DMSO (100%). Difference estimates in survival were compared using a *p* < 0.05 log-rank test.

The in vitro toxicity results showed no toxicity to human platelets at the tested AAPO concentrations (50, 100, 200, 400, and 800 μg/mL) (Figure 1A). There was no significant difference between concentrations and the negative control (NC), and the percentage range of platelet viability obtained fell between 97.4% and 99.1%. The in vivo toxicity test using *G. mellonella* larvae confirmed the absence of systemic toxicity at the concentrations tested (50, 100, 400, and 800 μg/mL), being equivalent to the NC (Figure 1B), without high levels of melanization or larvae death.

These results corroborate the data found by Traesel and colleagues (2014) [17], who demonstrated through an in vivo model using Wistar rats, low acute and subacute (28 days) toxicity of AAPO on blood cells, suggesting toxicity only at high concentrations of AAPO (50% oral lethal dose higher than 2000 mg/kg).

### 3.3. Effect of AAPO on Human Platelet Aggregation

**Figure 2:**
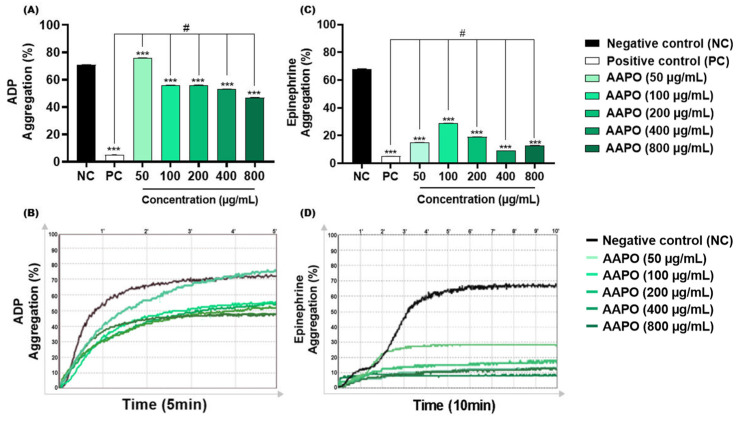
Effect of AAPO on platelet aggregation induced by ADP and epinephrine. Percentage (%) of platelet aggregation at different concentrations of AAPO (50, 100, 200, 400, and 800 μg/mL), induced by ADP (30 μM) (**A**,**B**) and epinephrine (5 μg/mL) (**C**,**D**) for 5 and 10 min, respectively; negative control—NC (DMSO 0.6%) and positive control—PC (Ticlopidine 10 μM). (***) The statistical difference with *p* < 0.001 compared to negative control (NC). (#) The statistical difference with *p* < 0.001 compared to positive control (PC).

ADP is concentrated in the dense granules of platelets, when released it binds to two purinergic receptors, P2Y12 and P2Y1 [36]. The first mediates the inhibition of adenylyl cyclase activity, activates phosphatidylinositol 3-kinase (PI3-K), and promotes the activation and amplification of platelet aggregation, forming stable thrombi [36]. On the other hand, P2Y1 leads to increased intracellular calcium, altered platelet shape, and reversible aggregation [37]. The results obtained in this study demonstrated significant inhibition of this aggregation pathway in AAPO-treated PRP, with the highest inhibitory percentage (34%) at a concentration of 800 μg/mL (Figure 2A,B).

Like ADP, AAPO showed antithrombotic action when epinephrine was used as an agonist. In addition to acting synergistically with other platelet agonists, epinephrine may assist in decreasing the concentration of cyclic adenosine monophosphate (cAMP) and activation of PI3-K, facilitating the thrombus formation process by binding to the adrenergic receptor (α2A) exposed on the platelet membrane [38]. Aggregation induced by this pathway was inhibited by more than 50% at all concentrations tested, with the highest percentage of inhibition (≈87%) in platelets treated with 400 μg/mL of bocaiúva pulp oil (Figure 2C,D).

No previous investigations on the antiplatelet effects of AAPO were found, making it difficult to compare the data with other studies. Nevertheless, antiaggregant effects have been previously reported in platelets treated with oil extract of the buriti fruit peel (*Mauritia flexuosa* L. F.), a palm species of the Arecaceae family, with 50% inhibitory concentrations (IC50) of 0.65 mg/mL for ADP and 0.93 mg/mL with collagen [39]. In our study, CI50 of AAPO against ADP-induced aggregation was 590 µg/mL (or 0.59 mg/mL), showing to be more potent than the oil extracts tested in those previous works. On the other hand, AAPO IC50 against epinephrine-induced aggregation was not possible to calculate, since all concentrations are capable of inhibiting more than 50% of platelet aggregation, showing an even more potent antiplatelet effect when compared to ADP.

However, the ability of oleic acid to modulate the action of receptors coupled to G proteins, mainly adrenoreceptors, and consequently its influence on adenylyl cyclase activity, has been previously reported [40]. In our study, we can observe that oleic acid is the most significant compound of AAPO (Table 1), thus, corroborating the antiplatelet activity of bocaiúva oil, especially when epinephrine is used as an agonist.

### 3.4. Effect of AAPO on Blood Coagulation

The anticoagulant activity of AAPO was analyzed by aTTP, which evaluates the integrity of the intrinsic and common coagulation pathways, and by PT, which evaluates changes in the coagulation factors of the extrinsic pathway [41].

Although prolongation in PT and aTTP was reported in the plasma of mice treated with flour from the mesocarp of a palm tree of the Arecaceae, *Orbignya phalerata* Mart. [42], AAPO in natura did not significantly increase PT time and aTTP at any of the concentrations tested (Table 2).

### 3.5. Platelet Activation

#### 3.5.1. Effects of AAPO on Platelet Surface P-Selectin Expression

P-selectin is a transmembrane protein stored in platelet alpha granules and Weibel-Palade bodies in endothelial cells [43]. Different platelet agonists and high shear stress contribute to the exteriorization of P-selectin in the cell membrane [44]. When bound to PSGL-1 (P-selectin-1 glycoprotein ligand) and platelet glycoprotein (Gp)Ib, it plays an important role in leukocyte and platelet adhesion and rolling, microparticle release, expression of monocyte tissue factor [43], and in the size and stabilization of thrombi mediated by the Gp IIb/IIa–fibrinogen interaction [22].

**Figure 3:**
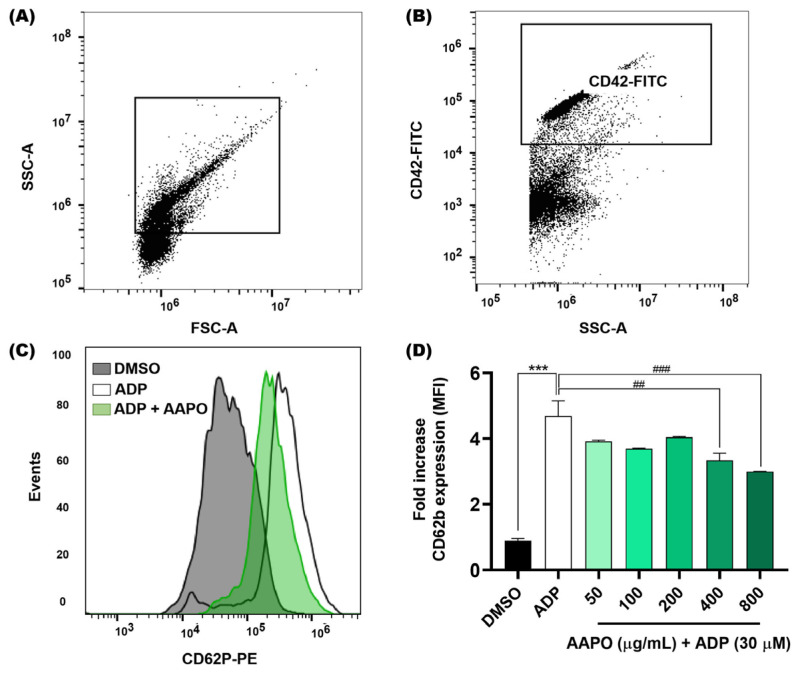
Expression of platelet surface P-selectin after exposure of platelets to AAPO. (**A**) Representative dot plots generated by FlowJo software. The gate shows the platelet population. (**B**) Representative dot plot showing a platelet-positive population (CD42b-FITC). (**C**) Representative histograms showing the activation of platelets incubated with AAPO and stimulated by ADP (30 μM) for 5 min. Activated platelets were labeled with CD42b-FITC (5 μL) and CD62P (CD62P-PE, 5 μL) and kept in the dark for 15 min. (**D**) Mean fluorescence intensity (MFI) of CD62P-PE expressed on the membrane of activated platelets treated with different concentrations of AAPO (50, 100, 200, 400, and 800 μg/mL). DMSO (0.6%): negative control; ADP (30 μM): positive control. Three independent experiments were performed. (***) The statistical difference with *p* < 0.001 compared to negative control. (##) The statistical difference with *p* < 0.01 and (###) statistical difference with *p* < 0.001 compared to ADP group.

We evaluated the expression of P-selectin in the membrane of activated platelets after exposure to AAPO (Figure 3) using the flow cytometry technique. As a result, we observed a significant reduction at concentrations of 400 and 800 μg/mL of AAPO. When compared to the results of platelet aggregation tests (Figure 2), it is observed that concentrations of 400 and 800 μg/mL present better results.

Furthermore, Fuentes and colleagues (2013) observed thrombin-induced inhibition of P-selectin expression in platelets treated with buriti oil extracts, where concentrations of 0.1 and 1 mg/mL inhibited approximately 18% and 29% of the expression of P-selectin, respectively [39].

#### 3.5.2. Content of ROS Produced by Platelets after Exposure to AAPO

**Figure 4:**
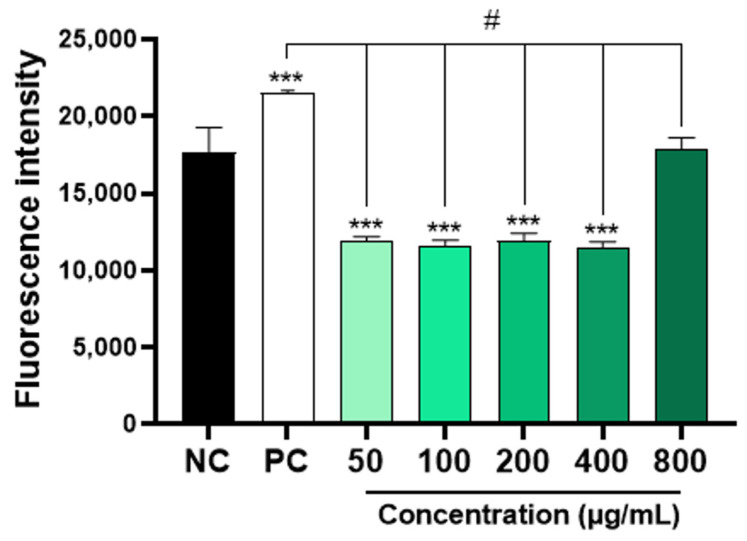
Intraplatelet content of ROS in AAPO-treated platelets. Intraplatelet ROS content after 30 min of incubation with DCFH-DA (10 µM), in platelets treated with 50, 100, 200, 400, and 800 μg/mL of *A. aculeata* pulp oil (AAPO) or controls; negative control—NC (DMSO 0.6%) and positive control—PC (hydrogen peroxide, H_2_O_2_). (***) The statistical difference with *p* < 0.001 compared to negative control (NC). (#) The statistical difference with *p* < 0.001 compared to positive control (PC).

The results obtained showed that concentrations of 50, 100, 300, and 400 μg/mL of AAPO significantly decreased (*p* < 0.001) the production of ROS when compared to the negative control (Figure 4). However, the same result was not observed in plasma treated with a concentration of 800 μg/mL. Physiologically, ROS are necessary for the maintenance of cellular function, while an imbalance between pro-oxidants and antioxidants generates a state of oxidative stress [45]. Increased platelet activation is directly associated with ROS generation and platelet adhesion receptor expression [2], so the use of ROS scavengers preserves platelet adhesion to collagen [46].

Recently, a higher total antioxidant capacity of bocaiúva pulp oil was reported in mice fed a high-fat diet [15]. In addition, the aqueous extract of dried leaves of *Acrocomia aculeata* (EA-Aa) revealed protective and hypoglycemic effects in type 2 diabetes promoted by polyphenols present in macaúba extracts (at concentrations of 125, 250, and 500 μg.mL^−1^) [16]. EA-Aa was able to protect against H_2_O_2_ at a concentration of 125 μg.mL^−1^ in human dermal microvascular endothelial cells and also in histological slices of liver, kidney, and aorta from Wistar and Goto-Kakizaki, whereas the antioxidant effect of EA-Aa on the vascular wall was also revealed in a tissue-specific manner in microvascular endothelial cell line represented by an improvement (≈22%) in cell viability [16]. Furthermore, our research group previously demonstrated that AAPO has a neuroprotective effect, being able to protect brain structures from oxidative damage induced by chronic restriction stress in Wistar rats, and effect attributable to the relatively high levels of α-tocopherol, β-carotene, and ascorbic acid found in AAPO [14].

Therefore, the data obtained here corroborate those found in the literature and highlight the present results regarding the antiaggregant potential of AAPO, which can be related to its promising ability to inhibit the production and release of ROS by platelets. This beneficial effect, as well as those demonstrated in related studies, is mainly attributable to the high concentration of compounds that possess a significant antioxidant capacity present in the macaúba fruit.

## 4. Conclusions

AAPO showed no toxicity in vitro or in vivo. It significantly inhibited platelet aggregation triggered by ADP and epinephrine in most of the concentrations tested, but it was unable to change coagulation parameters. In addition, AAPO decreased the expression of P-selectin on the platelet membrane and in the intraplatelet production of ROS, thus preventing platelet activation resulting in an antithrombotic effect.

## Figures and Tables

**Table 1:** Constituents identified from AAPO by GC-MS.

Peak	^1^RT (min)	Compound	Area (%)	^2^RI
1	6.66	Caproic acid	2.00	991
2	6.87	β-Myrcene	0.27	1002
3	8.05	β-phellandrene	1.73	1034
4	13.58	Caprylic acid	3.09	1186
5	14.21	Ethyl octanoate	0.16	1200
6	21.36	Capric acid	0.38	1375
7	29.24	Lauric acid	3.58	1573
8	36.35	Myristic acid	1.01	1768
9	39.82	Isobutyl phthalate	0.30	1870
10	41.47	β-Springene	0.55	1920
11	42.32	Palmitoleic acid	1.45	1947
12	43.16	Palmitic acid	21.51	1973
13	43.91	Ethyl palmitate	0.28	1996
14	49.19	Linoleic acid	0.24	2137
15	48.66	Oleic acid	51.25	2154
16	49.13	Ethyl oleate	8.45	2169
17	61.15	Hexacosane	0.77	2619
18	62.76	Heptacosane	0.14	2700
19	65.20	Squalene	2.37	2831
20	66.37	Nonacosane	0.46	2901

^1^RT: retention time; ^2^RI: retention indices on RTx-5MS capillary column.

**Table 2:** PT and aPTT coagulation parameters measured in human plasma treated with heparin and AAPO.

Sample ^1^	Blood Coagulation Test ^2^			
	PT		aPTT	
	Time (s)	IRN ^3^	Time (s)	Ratio ^4^
Normal control (Standard)	14 ± 0.32	-	28 ± 2.20	-
Negative control	15 ± 0.22	1.1	26 ± 1.53	0.9
AAPO (50 μg/mL)	16 ± 2.63	1.2	30 ± 0.62	1.1
AAPO (100 μg/mL)	17 ± 1.57	1.2	28 ± 1.24	1.0
AAPO (200 μg/mL)	17 ± 1.22	1.2	26 ± 3.93	0.9
AAPO (400 μg/mL)	17± 1.17	1.3	27 ± 1.12	1.0
AAPO (800 μg/mL)	16 ± 0.76	1.2	24 ± 2.08	0.8
Heparin (17 IU/mL of blood)	>100	-	>100	-

^1^ Oil from *A. aculeata* pulp (AAPO) at different concentrations (50, 100, 200, 400, and 800 μg/mL); negative control (vehicle, 0.6% DMSO) and heparin (positive control). ^2^ Prothrombin time (PT) and activated partial thromboplastin time (aPTT) values in seconds (s) measured in AAPO-treated human plasma and expressed as mean ± SD. ^3^ International normalized ratio (INR) calculated based on PT results, used to monitor the effectiveness of anticoagulants. ^4^ Relationship between the time values of concentrations and the time (s) of the controls.

## Data Availability

The data presented in this study are available on request from the corresponding author. The data are not publicly available due to privacy.
